# Metal and Microelement Biomarkers of Neurodegeneration in Early Life Permethrin-Treated Rats

**DOI:** 10.3390/toxics4010003

**Published:** 2016-01-29

**Authors:** Cinzia Nasuti, Stefano Ferraro, Rita Giovannetti, Marco Piangerelli, Rosita Gabbianelli

**Affiliations:** 1 Unit of Pharmacology, School of Pharmacy, University of Camerino, Via Gentile III da Varano, 62032 Camerino, MC, Italy; cinzia.nasuti@unicam.it; 2 Unit of Chemistry, School of Science and Technology, University of Camerino, Via S. Agostino 1, 62032 Camerino, MC, Italy; stefano.ferraro@unicam.it (S.F.); rita.giovannetti@unicam.it (R.G.); 3 Computer Science Division, School of Science and Technology, University of Camerino, Via del Bastione 1, 62032 Camerino, MC, Italy; marco.piangerelli@unicam.it; 4 Unit of Biochemistry and Molecular Biology, School of Pharmacy, University of Camerino, Via Gentile III da Varano, 62032 Camerino, MC, Italy

**Keywords:** permethrin, early life exposure, rat, hair, metals, microelements

## Abstract

Hair is a non-invasive biological material useful in the biomonitoring of trace elements because it is a vehicle for substance excretion from the body, and it permits evaluating long-term metal exposure. Here, hair from an animal model of neurodegeneration, induced by early life permethrin treatment from the sixth to 21th day of life, has been analyzed with the aim to assess if metal and microelement content could be used as biomarkers. A hair trace element assay was performed by the ICP-MS technique in six- and 12-month-old rats. A significant increase of As, Mg, S and Zn was measured in the permethrin-treated group at 12 months compared to six months, while Si and Cu/Zn were decreased. K, Cu/Zn and S were increased in the treated group compared to age-matched controls at six and 12 months, respectively. Cr significantly decreased in the treated group at 12 months. PCA analysis showed both a best difference between treated and age-matched control groups at six months. The present findings support the evidence that the Cu/Zn ratio and K, measured at six months, are the best biomarkers for neurodegeneration. This study supports the use of hair analysis to identify biomarkers of neurodegeneration induced by early life permethrin pesticide exposure.

## 1. Introduction

Hair is a non-invasive biological material useful for biomonitoring of heavy metals and microelements because it is a vehicle of substance excretion from the human body [1], and it permits evaluating long-term exposure of metals and microelement accumulation.

Heavy metals in hair are up to 10-fold higher than the levels found in blood or urine [2]. This is mainly due to the presence of cystine and metallic cations that form bonds with the sulfur of the matrix of hair keratin. For this reason, it represents an attractive choice for occupational and environmental surveys. Moreover, hair samples have the advantage of being a stable matrix, and its collection, transport and storage are very simple. Besides, it is a biological material better accepted by the population than blood samples, and it is available for repeated determinations over time. The Environmental Protection Agency (EPA) has accepted the value of human hair as a matrix for environmental monitoring. 

A correlation between neurodegeneration (*i.e.*, idiopathic Parkinson’s disease (PD)) and early onset of the disease has been observed in exposed occupational subjects with no family history of disease among their first-degree relatives [3]. At the same time, epidemiological investigations and studies in laboratory animals have shown evidence of the role of metals, neurotoxicants and pesticides in the onset of PD [3,4,5,6,7,8,9,10,11,12,13,14]. 

However, the surplus of heavy metals and the deficit of microelements in the body may not only be related to the extent of exposure, but also to the activity of enzymes responsible for their detoxification. Increased synthesis of metal-binding proteins, such as metallothioneins, in response to elevated levels of metals is the body’s primary defense against poisoning. These molecules are rich in thiol ligands, which allow high-affinity binding with cadmium, copper, silver and zinc, among other elements. Other proteins involved in both heavy metal transport and excretion, through the formation of ligands, are ferritin, transferrin, albumin and hemoglobin. Moreover, heavy metals can be detoxified by enzymes of phase I and II. The latter can work differently, according to their own polymorphism and epigenetic regulation, leading to accumulation of heavy metals or to depletion of essential trace elements in the body [15]. According to this, environmental exposure to metals and neurotoxicants can have a different impact on cohabiting partners, due to their different enzymatic activities [16,17].

Taking into account this evidence, the aim of this study was to evaluate the level of metals and microelements in neck hair from permethrin (PERM)-treated rats exposed to the pesticide during early life. Two time points were chosen for assessment in rats, at six and 12 months of age, because at this age, animals showed dopamine, Nurr1, oxidative stress and behavioral abnormalities similar to those observed in Parkinson’s-like diseases [8,9,10,11,12,13,14]. Data were discussed considering the role of trace elements and the biomarkers previously measured in this animal model of neurodegeneration. 

The goal of the study was to look for hair biomarkers to be used as early signs of neurodegeneration as an alternative to neuronal biomarkers that were already defined in the present animal model (Nurr1, dopamine, GSH, *etc.*) [8,9,10,11,12,13,14]. Besides, an impairment of metals and microelements in the hair following early life permethrin treatment could highlight the toxicity of this pesticide underlining the interplay between early life exposure and the long-term effect of this pesticide’s wide use today. In the future, the translation of these results in humans may permit screening neurodegenerative diseases in the early phase.

## 2. Experimental Section

### 2.1. Materials

All reagents used for this study were of analytical grade. Technical grade (75:25, trans:cis, 94% purity) 3-phenoxybenzyl-(1R,S)-cis,trans-3(2,2dichlorovinyl)2,2dimethylcyclopropanecarboxylate, permethrin, was a generous gift by Antonio Stefanini of ACTIVA, Milan, Italy.

### 2.2. Rats

Wistar rats aged about 90 days weighing 250–270 g were purchased from Charles River (Calco, LC, Italy). The rats were housed in plastic cages in a temperature-controlled room (21 ± 5 °C) and fed with a laboratory diet and water *ad libitum*. The light/dark cycle was from 7 a.m. to 7 p.m. The rats employed were treated according to the European Directive (2010/63/EU) related to the protection of animals used for scientific studies. Rat pups born in our laboratory from primiparous dams were used in the study, and the parturition day was set as Post Natal Day 0 (PND0). On PND1, all litters were checked for the presence of gross abnormalities, sexed and weighed. Male pups (*n* = 24) were assigned to a dam until weaning (PND21). No cross-fostering was employed. At 2 days of age, litters were casually assigned to two experimental groups, named the control and treated groups.

### 2.3. Treatment and Experimental Design

This study was performed using 9 rats (*n* = 5 control and *n* = 4 PERM-treated) to investigate metal and microelement contents in hair at two time points, 6 and 12 months of age.

PERM was solubilized in corn oil, and the animals were treated by gavage with the pesticide at a dose of 1/50 of LD_50_ corresponding to 34.05 mg/kg (Agency for Toxic Substance and Disease Registry, 2005). The dosage was chosen considering that NOAEL (no observed adverse effect level) for PERM is 25 mg/kg. The PERM group received the pesticide daily in the morning from PND6 to PND21. 

The control group was administered with vehicle (corn oil, 4 mL/kg) on a similar schedule. The volume of solutions to be administered was adjusted daily based on body weight. At the age of 6 and 12 months, neck hair (length = 2 cm) close to the scalp from the same animal was collected and used for the analysis of metals and microelements. 

### 2.4. Hair Analysis

Before collection of hair, animals were cleaned with water-soaked paper towel and dried with paper towel. 

Forty seven heavy metals and microelements (Li, Be, B, Na, Mg, Al, Si, P, S, K, Ca, Ti, V, Cr, Mn, Fe, Co, Ni, Cu, Zn, Ga, Ge, As, Se, Rb, Sr , Zr, Nb, Mo, Ru, Rh, Pd, Ag, Cd, Sn, Sb, Te, Cs, Ba, Hf, Ta, W, Re, Pt, Au, Hg, Tl, Pb, U) were analyzed by the ICP-MS technique in hair of PERM and control rats. This validated method is widely used to analyze heavy metals and microelements [18,19]. Hair samples were cut into small pieces using clean stainless steel scissors. About 60 ± 7 mg were transferred into tarred, labeled centrifuge tubes, and the exact weight was recorded. To each sample digestion tube, 5 mL of reagent-grade nitric acid (HNO_3_) were added as a mineralizing solution. Samples were incubated for 10 min and then subjected to acid microwave digestion using a microwave vessel (Berghof Speedwave 4, Berghof, Eningen, Germany) order to solubilize the elements of interest [20,21]. After the mineralization process, the samples were cooled down to ambient temperature, and then, the solution was transferred to 10-mL tubes and filled up with reagent grade water Type 1. One milliliter of solution was transferred to a tube and diluted 10 times with reagent grade water Type 1 in order to decrease the acid concentration. The solution was analyzed for amounts of mineral elements and trace metals by inductively-coupled plasma mass spectrometry (ICP-MS, Agilent Technologies, Santa Clara, CA, USA). Sample results were quantified by a multiple calibration curves for all elements, and finally, they were checked by comparison with certified reference materials hairs (ERM DB001, GBW 07601) treated in the same modality.

### 2.5. Statistical Analysis

Exploring data to find hidden relationships, to cluster them or reduce the dimensionality of the dataset, is a first step towards good data analysis. In order to achieve one of these targets, a very simple, but powerful technique, called principal component analysis (PCA), was used. PCA is an unsupervised statistical procedure based on linear algebra, in particular on singular value decomposition (SVD) that transforms your dataset, using an orthonormal transformation, from a highly correlated dataset to a linearly uncorrelated one. Basically, applying the PCA algorithm, we answer the following question: is there another basis, which is a linear combination of the starting basis, that better describes our dataset? If so, the new coordinates are the eigenvectors of the transformed dataset and are called principal components (PCs); PCs that better express our dataset are the ones with the largest possible variance. The PCA analysis was performed using the software R (Free software R (Version 3.2.2, R Core Team) under GNU General Public License, www.r-project.org) under the GNU free license.

Hair samples were analyzed individually, and outcomes were presented in parts per million (ppm). Results were expressed in ppm as the mean ± SEM. Statistical analysis was carried out using the program Statistica 8.0 (StatSoft Italy Srl, Vigonza, PD, Italy, 2007). The outcomes were analyzed with parametric (ANOVA) or non-parametric (Kruskal–Wallis) tests according to their normal or non-normal distribution. Repeated measures two-way ANOVA followed by the *post hoc* Newman–Keul test were used for Si, S, K and Cu/Zn with a normal distribution, while the Kruskal–Wallis test was employed for all of the others. Differences were considered significant at a *p*-value <0.05.

## 3. Results

### 3.1. General Findings

Early life PERM-treated rats compared to the control group at both time points did not show any significant change in body weight (Figure S1).

### 3.2. PCA Analysis

Table 1 contains the values of the eigenvalues associated with the principal components, *i.e.*, the eigenvectors. According to the numeric values, we chose to reconstruct our dataset using only the first two PCs. 

**Table 1:** Eigenvalues associated with the principal components after elaboration of the whole dataset considering both the control (*n* = 5) and permethrin (PERM) (*n* = 4) groups.

Principal Component	Eigenvalue
1	6.9296
2	1.5313
3	1.0504
4	0.5982
5	0.2797
6	0.2719
7	0.1736
8	0.1318
9	0.0211
10	0.0124
11	8.56 × 10^−8^

**Figure 1:**
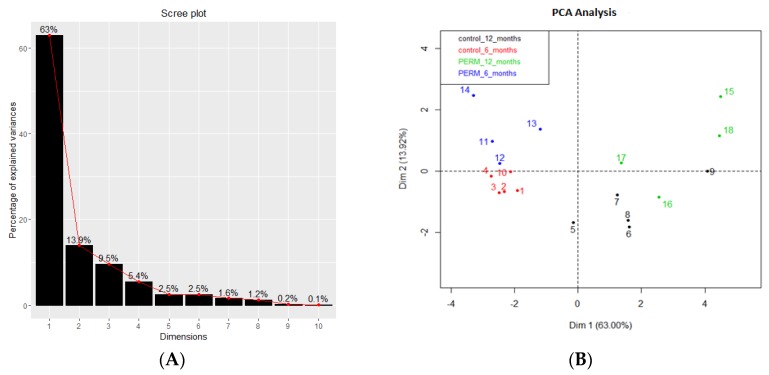
Scree plot (**A**) and Principal Component (PCA) plot (**B**) based on all microelements from hair of control and Permethrin (PERM)-treated rats at six and 12 months of age.

The Scree plot in Figure 1A, shows the contribution of the first ten eigenvalues: the first three eigenvalues are responsible for more than 85% of the explained variance (the detailed values are in Table 1). In the PCA plot (Figure 1B), it is possible to observe four different clusters corresponding to control and PERM at six and 12 months. The temporal distinction of clusters (red and blue marks against black and green ones) is clear; in addition, a clear separation between control and PERM groups can be observed at six months. On the contrary, at 12 months, the difference between both groups (black and green marks) cannot be precisely detected. 

### 3.3. Metals and Microelements in Hair

**Figure 2:**
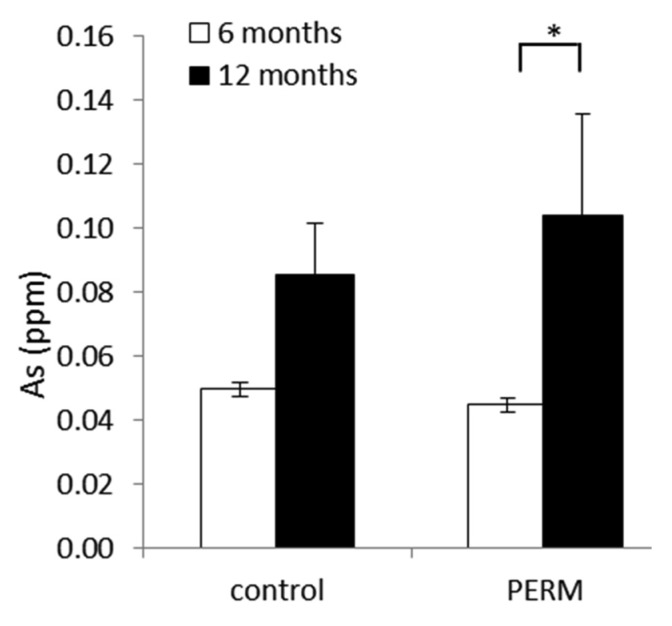
Analysis of As in hair from early life PERM-treated rats and control ones at six and 12 months of age. Data are expressed as the mean values ± SEM. * *p* < 0.05.

Shapiro–Wilk’s test was applied to each microelement for testing the normality of the data. The data of Na, Si, S, K, Se, Na/K and Cu/Zn were not normally distributed, and their values were *w* = 0.91 (*p* = 0.11), *w* = 0.95 (*p* = 0.51), *w* = 0.93 (*p* = 0.21), *w* = 0.94 (*p* = 0.30), *w* = 0.95 (*p* = 0.53), *w* = 0.96 (*p* = 0.55) and *w* = 0.90 (*p* = 0.058), respectively. Data normally distributed were computed by two-way ANOVA for repeated measures, whereas data not normally distributed were analyzed by the non-parametric Kruskal–Wallis test. Rats exposed to PERM during early life (only 15 days of treatment) showed a significant increase of As at 12 months compared to six months (*H* [3,18] = 12.72, *p* = 0.0053), while no statistical differences were observed between treated and control groups at all time points (Figure 2).

**Figure 3:**
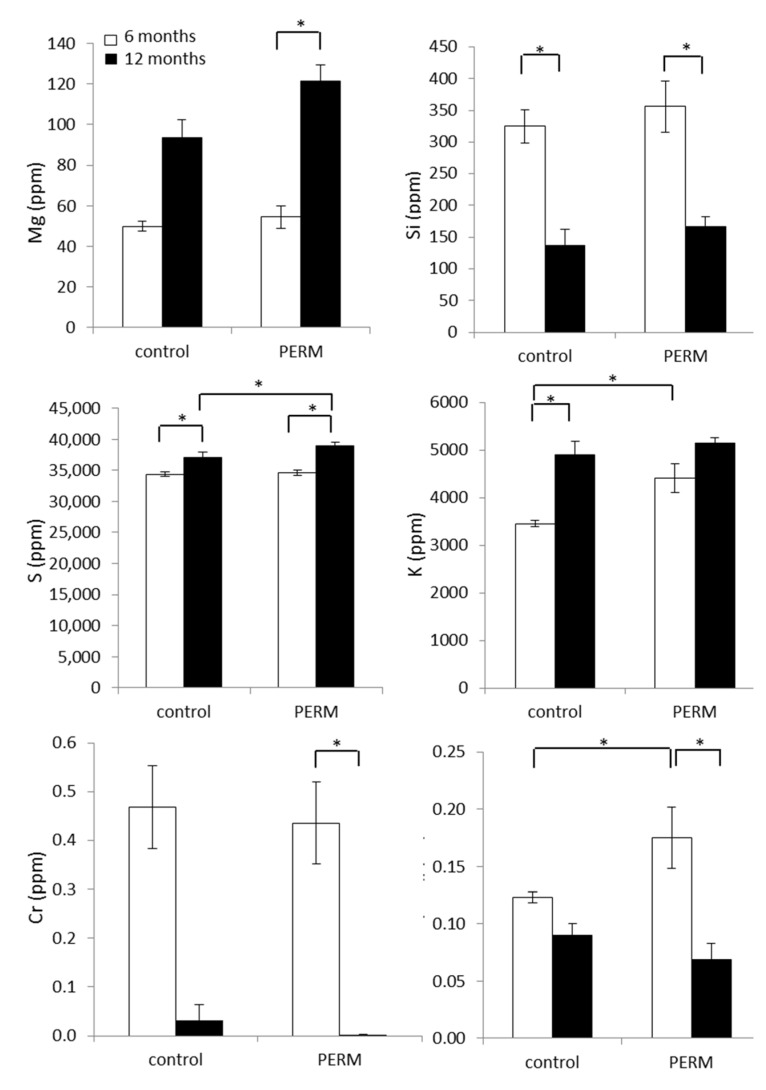
Analysis of microelements in hair from early life Permethrin (PERM)-treated rats and control ones at six and 12 months of age. Data are expressed as the mean values ± SEM. * *p* < 0.05.

Other microelements were significantly modified by early life exposure to PERM (Figure 3). Within the PERM group, Mg was increased at 12 months of age compared to six months of age (*H* [3,18] = 13.49, *p* = 0.0037), as well as S was higher than that measured in the matched control group at the same age as revealed by a significant treatment × time interaction, *F* [1,7] = 31.69, *p* = 0.00079.

K was significantly higher in the treated group at six months compared to the value measured in the control one, while it was unchanged at 12 months. In addition, the control group showed a higher level of K at 12 months with respect to six months (*F* [1,7] = 8.61, *p* = 0.021).

A decreased level of Cr was measured in the PERM group at 12 months, while the same outcome was not significant in the control group (*H* [3,18] = 14.22, *p* = 0.0026). The Cu/Zn ratio was increased in the PERM group at six months of age with respect to the control one, while the ratio decreased significantly within the PERM-treated group at 12 months (*F* [1,7] = 14.65, *p* = 0.006), as revealed by a significant treatment × time interaction.

**Figure 4:**
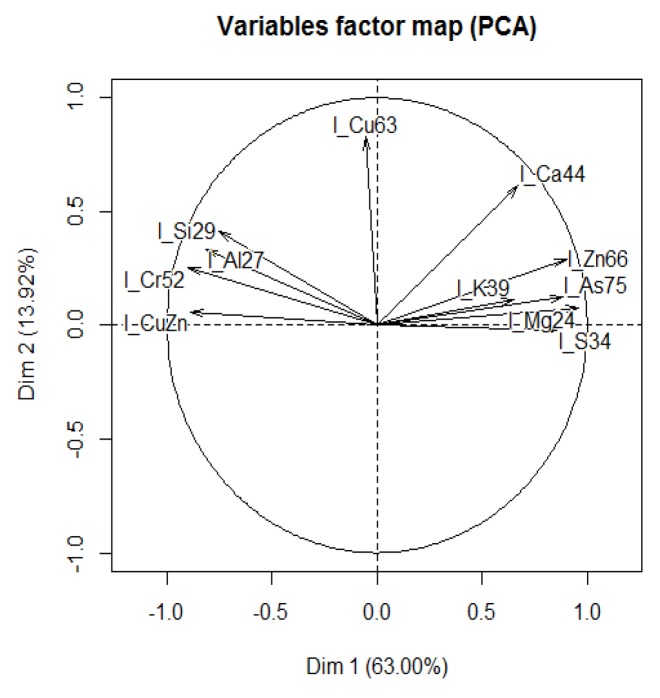
Principal Component (PCA) analysis on metal and microelements that were significantly changed.

PCA analysis on metals and microelements that were significantly changed showed two clusters (Figure 4). In particular, the variable factor map indicates that PC1 (Dim1) was correlated with all microelements except Cu. All microelements on the right of the picture were negatively correlated with those in the left of the map, except for Cu, associated with PC2 (Dim2), which was negatively correlated with Ca. 

Table 2 shows some of the more representative metals and microelements measured in hair of rats at both time points, six and 12 months. Although some of these data (*i.e.*, Na, Al, P, Ca, *etc.*) have a different trend in the two groups and time points, the statistical analysis of the data cannot permit defining significant differences between groups.

**Table 2:** ICP-MS analysis of the main metals and microelements contained in the neck hair of rats, exposed to Permethryn (PERM) during early life. Data are the means ± SEM at two time points.

Metals/Microelements	Control (ppm)	PERM (ppm)	Control (ppm)	PERM (ppm)
	6 Months		12 Months	
Na	658.134 ± 33.84	715.742 ± 72.27	909.209 ± 96.57	785.487 ± 71.05
Al	117.362 ± 37.24	521.173 ± 222.12	16.769 ± 5.56	29.280 ± 12.16
P	355.566 ± 5.94	393.904 ± 27.39	344.279 ± 18.53	382.025 ± 8.22
Ca	262.810 ± 16.53	347.534 ± 75.02	387.015 ± 169.62	1213.493 ± 503.90
Mg	49.862 ± 2.34	54.471 ± 9.00	93.547 ± 5.44	121.550 * ± 7.79
Cr	0.469 ± 0.09	0.436 ± 0.08	0.032 ± 0.03	0.000 ± 0.00
Mn	0.692 ± 0.04	0.904 ± 0.08	0.529 ± 0.09	0.983 ± 0.43
Fe	19.626 ± 3.01	19.494 ± 1.31	11.031 ± 0.83	19.308 ± 5.06
Ni	0.331 ± 0.15	3.456 ± 2.92	0.366 ± 0.11	0.544 ± 0.12
Cu	13.358 ± 0.42	18.625 * ± 2.43	13.371 ± 0.75	16.065 ± 0.58
Zn	108.667 ± 1.67	107.232 ± 30.36	162.719 ± 2.13	271.370 ° ± 64.67
Se	0.175 ± 0.01	0.158 ± 0.01	0.209 ± 0.03	0.219 ± 0.03
Rb	2.631 ± 0.08	3.365 ± 0.27	4.309 ± 0.15	4.766 ± 0.25
Sr	0.261 ± 0.02	0.603 ± 0.08	0.249 ± 0.10	0.677 ± 0.27
Ag	0.016 ± 0.01	0.032 ± 0.02	0.021 ± 0.00	0.086 ± 0.05
Cd	0.001 ± 0.00	0.001 ± 0.00	0.014 ± 0.01	0.030 ± 0.03
Sn	0.058 ± 0.02	0.031 ± 0.01	0.031 ± 0.02	0.028 ± 0.03
Sb	0.003 ± 0.00	0.006 ± 0.00	0.020 ± 0.01	0.040 ± 0.03
Pt	0.003 ± 0.00	0.003 ± 0.00	0.012 ± 0.01	0.025 ± 0.03
Au	107.047 ± 1.88	105.883 ± 0.96	0.099 ± 0.02	0.128 ± 0.06
Hg	0.052 ± 0.01	0.031 ± 0.00	0.057 ± 0.01	0.039 ± 0.03
Pb	0.105 ± 0.02	8.744 ± 8.66	0.106 ± 0.05	0.284 ± 0.08
Na/K	0.191 ± 0.01	0.162 ± 0.01	0.186 ± 0.02	0.152 ± 0.01
Na/Mg	13.261 ± 0.69	13.168 ± 0.58	10.252 ± 1.72	6.445 ± 0.29
Ca/K	0.076 ± 0.00	0.077 ± 0.01	0.083 ± 0.04	0.233 ± 0.10
Ca/Mg	5.269 ± 0.21	6.262 ± 0.81	3.895 ± 1.34	9.387 ± 3.56

* *p* < 0.05 *vs.* the age-matched control group; °*p* < 0.05 *vs.* the same group at 6 months.

## 4. Discussion

The animal model of neurodegeneration, presented here, is an extensively-studied model characterized by the typical features of Parkinson’s-like disease, induced by 15 days of PERM treatment during early life [8,9,10,11,12,13,14]. The interest toward PERM depends on its larger use as an insecticide in agriculture, in houses and as a fungicide for wood maintenance [22]. Its neurotoxicity was observed in rats exposed to PERM in early life, and in their untreated offspring, underlining the intergenerational effect of this toxic [23]. In fact, because of its high lipophilicity, PERM can easily cross the blood brain barrier, and it can be accumulated into the brain a long time after exposure [24] Data on 300-day-old rats, treated with PERM during early life, showed a significant decrease of Ca in striatum, hippocampus and plasma, together with a low level of superoxide dismutase in plasma and an increase of lipid peroxidation in striatum [8]. A low level of Ca and a high level of Na and NO in plasma were measured in the same animal model of 500-day-old rats [10]. Na excretion in urine of 500-day-old rats was significantly increased compared to the control group [10]. 

While metals and microelements in blood or urine are an index of recent exposure, their unbalance in hair represents a parameter correlated with long-term exposure. Outcomes, here reported on hair, showed a significant increase of As in hair of 12-month-old rats. Since it is a toxic metalloid used in the preparation of pesticides, it can be measured in water, food, soil and air [25]. This toxic metalloid was previously reported as increased with aging in hair of rats [26]; it has been reported to increase the occurrence of diabetes and cardiovascular disease [26]. Moreover, it can induce also intergenerational damage because of its capacity to lead to DNA methylation and histone modifications [27]. It should be underlined that also another metalloid of the same group, the XV of the periodic table of elements, Sb and the nonmetal P tend to have higher values in the treated group compared to those in the control one at both time points (Table 2). It should be considered that elements from the same group of the periodic table might be exchanged in the body because of their similar properties according to the requirements of the organism; As and P have similar physical and chemical characteristics, thus As can be used in place of P if the body requires P and the latter is deficient. In this context, it should be underlined that the animal model, presented here, showed alteration in the energy metabolism, since PERM treatment was able to lead to unbalanced activity in mitochondria complex I and a significant reduction of long-chain fatty acid transport 1, a protein required to transport long chain fatty acids into mitochondria for their oxidation [28,29].

Si, a metalloid of the XIV group, was significantly decreased in PERM and control groups at 12 months. Si is a component of both connective tissues and intracellular space in neurons, and it has a key role in the developmental process in young animals and humans [30]. Here, the significant decrease of Si cannot represent a biomarker of damage, because the same decrease was measured in both groups with aging.

S, a non-metal belonging to the XVI group, was increased with aging, and its content was highest in the treated group compared to the control group in 12-month-old rats. Sulfur is used in the preparation of insecticides and fungicides, but it is also an important component of amino acids (*i.e.*, cysteine, methionine taurine and homocysteine), as well as a key element for enzymes as complex I–III of the respiratory chain. Here, the increased level across time could be explained considering two speculative hypothesis: firstly, S could be more present to counterbalance the lower value of Se, an element of the same group, which resulted in being lower at six month of age in rats, according to the low Se-dependent glutathione peroxidase activity previously measured in this animal model [12]; secondly, S could be increased in the treated group at 12 months compared to the age-matched one, as a compensatory process to complex I inhibition induced by PERM, as previously reported [28].

K was increased in the PERM group compared to that in the control one, at six months of age. This alkaline metal has been reported higher together with Na, an element of the same I group, in aged rats affected by cardiac disease and renal failure [26]. These data were in agreement with our previous data showing increased Na in plasma of PERM-treated rats together with an impairment of catecholamine [10]. Moreover, since K regulates neuronal excitability through membrane repolarization, unbalanced K intracellular concentration has been connected with neuronal disorders [31]. In neurons, K can modulate apoptotic enzyme activities, and a low intracellular K concentration has been associated with a more feasible condition for a physiologic apoptotic process [31]. 

The decrease of Mg has been associated with inflammation and oxidative stress during aging [32]. Here, we reported an increase of Mg in the PERM group at 12 months. This outcome seems to be in contrast with the literature on aging; however, it can be considered that a high level of Mg was associated with high K and/or low Ca levels [32]. Although Ca was not significantly changed in hair, in our previous studies, on the same animal model, the Ca plasma level was decreased in adults and old age [10]. 

It is interesting to note the decrease of Cr in the PERM group at 12 months. Cr seems to be correlated with sugar metabolism, and a decrease of Cr has been associated with a deficit of insulin and perturbation of glycemic control [33]. As reported above, the animals treated in early life with PERM presented an unbalance in the energy metabolism related to alteration in lipid catabolism [10,29]. Moreover, recent data on myotubes exposed to PERM showed that this pesticide was able to reduce insulin-stimulating glucose uptake and adipogenesis in adipocytes [34].

The Cu/Zn ratio is associated with inflammation [35]. Here, PERM-treated rats at six months of age presented a significantly higher ratio of Cu/Zn compared to the control group, due to the elevated Cu level in the PERM-treated group. Cu has a key role as cofactor of many enzymes and proteins; its excess is associated with oxidative reactions and with the regulation of the iron level in brain; Cu excess is associated with brain dysfunction [35]. Moreover, a significant decrease of the Cu/Zn ratio at 12 months of age has been observed in the treated group due to a significant increase of Zn (Table 2). Zn is a cofactor of many proteins, and it has a key role in several physiological activities [36]. The high level of Zn is neurotoxic, and it has been linked to neurodegeneration [37]. Zn interacting with NMDA-type glutamate receptors regulates postsynaptic excitability, and it increases the expression of Ca channels. Furthermore, a high level of Zn can inhibit mitochondrial respiration, leading to energy depletion together with the production of reactive oxygen species [36,37]. 

## 5. Conclusions

Exposure to PERM in early postnatal life leads to metal and microelement alterations detectable in the hair of rats. This study conducted on rats exposed to the same conditions (diet, life style and environment) has the advantage to eliminating these discriminating factors that cannot be fixed in human studies.

PCA analysis of metals and microelements showed two clusters: Zn, AS, K, Mg and S were negatively correlated with Si, Cr, Cu/Zn and Al, while Cu was negatively correlated with Ca. A significant increase of As, Mg, S and Zn was measure in the permethrin-treated group at 12 months compared to six months, while Si and Cu/Zn were decreased. K, Cu/Zn and S were increased in the treated group compared to the age-matched controls at six months and 12 months, respectively. Cr resulted in being significantly decreased in the treated group at the second time point. 

PCA analysis showed the best difference between treated and age-matched control groups at six months. The present findings support the evidence that the Cu/Zn ratio and K, measured at six months, are the best biomarkers for neurodegeneration. 

This study represents the first step of a larger study aimed to clarify the role of metals and microelements in the neurodegenerative process induced by early life PERM exposure. Furthermore, the impairment of metals and microelements in the hair following early life permethrin treatment highlights the toxicity of permethrin pesticide, underlining the interplay between early life exposure and long-term effects. The identification of these unbalanced elements as biomarkers of neurodegeneration could represent a new way to screen which microelements can be associated with neuronal disorder consequent to PERM exposure during early life.

## Supplementary Files

Supplementary File 1

## Abbreviations

The following abbreviation sare used in this manuscript:

ICP: inductively-coupled plasma mass spectrometry
PCA: Principal component analysis
PERM: Permethrin
PC: Principal Component
DIM: Dimention
