# Supplementary material for: Metal and Microelement Biomarkers of Neurodegeneration in Early Life Permethrin-Treated Rats
Source: Toxics. 2016 Jan 29;4(1):3. doi: 10.3390/toxics4010003 (PMC5606634; doi:10.3390/toxics4010003)
Supplement: Supplementary File 1 [file toxics-04-00003-s001.pdf]

Supplementary Materials

Cinzia Nasuti, Stefano Ferraro, Rita Giovannetti, Marco Piangerelli and Rosita Gabbianelli

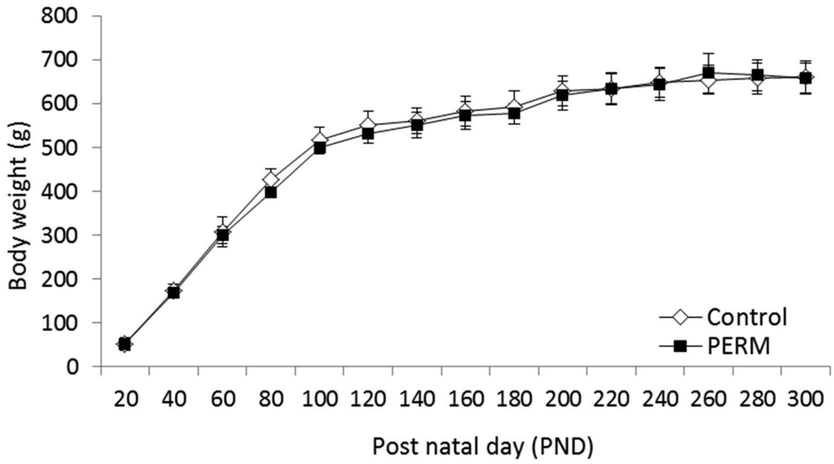

Figure S1. Rat body weight in control and PERM groups.
